# Effect of Glycerol on an N-Vinylpyrrolidone-Based Photopolymer for Transmission Holography

**DOI:** 10.3390/polym13111754

**Published:** 2021-05-27

**Authors:** Huishi Pi, Weiping Li, Zhiwei Shi, Haining Chen, Xiaoyu Jiang

**Affiliations:** 1 School of Materials Science and Engineering, Beihang University, Beijing 100191, China; pihuishi@163.com (H.P.); STone170930@163.com (Z.S.); chenhaining@buaa.edu.cn (H.C.); 2 Department of Information Communication, Army Academy of Armored Forces, Beijing 100072, China

**Keywords:** transmission holography, photopolymer, polymerization rate, diffusion rate, diffraction efficiency

## Abstract

N-vinylpyrrolidone (NVP) has a large molecular structure, so it is difficult to diffuse during holographic recording, especially at low spatial frequencies. We used glycerol to promote the diffusion of NVP, and successfully improved the holographic performance of the photopolymer at low spatial frequencies. As the concentration of glycerol increases, the holographic performance first increases and then remains stable. The optimal concentration of glycerol is 0.21 mol/L. At this concentration, the maximum diffraction efficiency of the photopolymer is 84%, the refractive index modulation is 1.95 × 10^−3^, and the photosensitive sensitivity is 7.91 × 10^−4^ cm^2^/mJ. Compared with the control group, the maximum diffraction efficiency, maximum refractive index modulation and photosensitivity at low spatial frequencies (800 lp/mm) have increased by 11.19 times, 4.69 times and 1.71 times, respectively. Using the optimized photopolymer for transmission holographic recording and reproduction, we have obtained a clear and bright transmission hologram. The photopolymer modified with glycerol is expected to be applied to the fields of holography, diffractive optics, and so on.

## 1. Introduction

A photopolymer for holography is a form of light-responsive material based on polymerization reaction, which has the advantages of high diffraction efficiency, high spatial resolution and a simple preparation process. It has broad application prospects in the fields of sensors [1,2,3,4], diffractive optical elements [5,6,7,8,9], holographic interferometry [10,11], displays [12,13] and holographic recording materials [14,15]. The photopolymer mainly contains film formers, photosensitizers, photoinitiators and polymerizable monomers. According to different solvents, photopolymers can be divided into two main categories: (1) oil-soluble photopolymers that use organic solvents to dissolve film-forming agents, and (2) water-soluble photopolymers that use water as a solvent. Compared with oil-soluble photopolymers, water-soluble photopolymers have the advantages of being non-toxic, environmentally friendly and low in cost. Nowadays, the development of such photopolymers is an increasingly active research area.

Holography is an important application field. The image produced by a hologram has the advantage of a 3D effect and realistic depth perception. Holography mainly has two recording methods, reflective and transmissive [16,17]. Transmission holography is the main method to obtain holograms of large scenes, so it has greater application prospects. During the actual transmission holographic recording process, the angle between the object beam and the reference beam is often required to be kept within a reasonably small range (less than 40°) to achieve a better interference pattern [17]. In other words, to obtain high-quality transmissive holograms, photopolymer materials are required to have excellent holographic performance at lower spatial frequencies. As a common polymerizable monomer, NVP has been successfully used in the preparation of water-soluble polyvinyl alcohol system photopolymers [16]. NVP has a high refractive index (*n* = 1.512). When NVP is used in a polyvinyl alcohol system, the saturation refractive index modulation reaches 1.2 × 10^−2^, which makes it an excellent candidate for preparing photopolymers with high refractive index modulation. However, NVP has a five-membered ring substituent, and its molecular weight is much larger than that of acrylamide [16,18], which makes the diffusion of NVP slow during the holographic recording process. This is not conducive to the realization of holographic recording, especially long-distance diffusion holographic recording at low spatial frequency. The holographic performance of an NVP-photopolymer at low spatial frequency has become an objective obstacle to its application in transmission holography. Therefore, improving the diffusion rate of NVP in photopolymers is necessary to improve the holographic performance of NVP-photopolymers at low spatial frequencies. Zhu Jianhua et al. found that monomers exhibit different diffusion rates in PVA films that have different glass transition temperatures [19]. The low glass transition temperature of the film-forming agent is conducive to the diffusion of the monomer. The most common method to change the glass transition temperature of a polymer is to add a plasticizer. Common plasticizers used for PVA modification mainly include water, ethylene glycol and glycerol [20,21,22]. Compared with water and ethylene glycol, glycerol has a significant advantage. Glycerol is not easy to volatilize during the film preparation process because of its high boiling point, which allows it to achieve a more stable plasticizing effect. Therefore, glycerol is an excellent choice for modifying polyvinyl alcohol. In addition, it has previously been shown to be responsible for significant improvements in holographic characteristics for similar polyvinyl alcohol systems [18].

This paper is based on NVP-based photopolymers and introduces glycerol as a modifier to improve the holographic properties of the photopolymers. A photopolymer with high diffraction efficiency, high refractive index modulation and high sensitivity is obtained. In addition, the effect of glycerol on the diffusion rate and polymerization rate of NVP in the material was studied. The formation process of the refractive index grating in the photopolymer, with or without glycerol, is discussed. The optimized NVP-photopolymer was used for transmission holographic printing. A clear and bright transmission hologram was obtained. The experimental results can provide effective guidance for the improvement of photopolymer performance.

## 2. Materials and Methods

### 2.1. Materials

First, water, Polyvinyl alcohol (Sigma Aldrich, Merck KGaA, Gernsheim, Germany), tetraiodofluorescein sodium (AR; Aladdin Biochemical Technology Co., Ltd. Shanghai, China), triethanolamine (AR; Aladdin Biochemical Technology Co., Ltd. Shanghai, China), *N*,*N*′-methylenebisacrylamide (AR; Aladdin Biochemical Technology Co., Ltd. Shanghai, China) and NVP (AR; Aladdin Biochemical Technology Co., Ltd. Shanghai, China) are dissolved in water at the mass ratio of 86:8:0.02:2:1:3 to obtain a photosensitive liquid. The above liquid was equally divided into six parts. Different concentrations of glycerol were added to obtain the final photosensitive liquid. The concentrations of glycerol are 0 mol/L, 0.035 mol/L, 0.07 mol/L, 0.14 mol/L, 0.21 mol/L and 0.28 mol/L, respectively. Then, the photopolymer photosensitive liquid obtained above is drawn and coated on a glass slide (76 mm × 25 mm). Finally, the photosensitive liquid is dried in the dark to obtain a uniform and transparent photopolymer. The ambient temperature is 20–25 °C, and the relative humidity is 40–70%. The thickness of the film layer, measured by a spiral micrometer, is about 110 μm.

### 2.2. Theory

**Figure 1:**
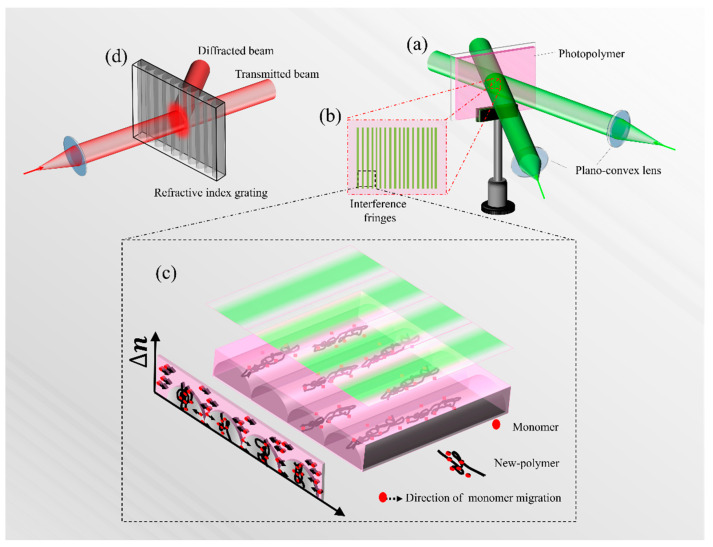
Schematic diagram of the recording and reproduction principle of photopolymer holography: (**a**) photopolymer exposure process; (**b**) interference fringes formed by object light and reference light; (**c**) formation process of refractive index grating inside photopolymer. (**d**) Reproduction of hologram.

Photopolymers are a type of light-responsive material based on polymerization reactions. The principle of holographic recording is shown in Figure 1. In the process of photopolymer holographic recording, the object light interferes with the reference light to form interference fringes, as shown in Figure 1a,b. When the interference fringes irradiate the photopolymer, the photosensitizer in the bright area (or constructive interference area) absorbs photons and generates free radicals to initiate polymerization. However, the photosensitizer in the dark area (or destructive interference area) has no photon absorption and cannot generate free radicals to initiate monomer polymerization. As the reaction progresses, a monomer concentration gradient will be formed between the light and dark areas in the photopolymer. Driven by the concentration gradient, the monomer in the dark area will diffuse to the light area to continue the reaction. A large amount of nascent polymer will be enriched in the bright area. The film-forming agent and a small amount of unreacted monomer remain in the dark area. Due to the difference in refractive index between the nascent polymer and the film-forming agent, a refractive index grating will be formed in the material. By converting the object information into the difference in internal component distribution and the difference in refractive index, the photopolymer achieves the goal of information recording, as shown in Figure 1c. When the recorded photopolymer is irradiated by a beam of light at a specific angle, the internal refractive index grating modulates the beam and causes diffraction. Then, the previously recorded object information will be reproduced in the form of diffracted light, as shown in Figure 1d.

The holographic properties of photopolymers are mainly characterized from three perspectives: diffraction efficiency, refractive index modulation, and photosensitivity. The diffraction efficiency, the macroscopic result of holographic performance, determines the brightness of the reproduced image under a specific light intensity: the higher the diffraction efficiency, the higher the brightness of the observed image. Improving the diffraction efficiency of photopolymers has always been the research direction of photopolymer performance optimization. The degree of Δn is a key parameter that reflects the light modulation ability of the photopolymer. The greater the refractive index modulation degree, the stronger the optical control ability of the refractive index grating inside the photopolymer. Photosensitivity is a parameter that reflects the response speed of the photopolymer to incident light. A photopolymer with high photosensitivity can complete holographic recording faster. In addition, it has greater tolerance to system errors such as platform shaking and airflow, which is more conducive to obtaining high-quality recording. The test method of photopolymer holographic performance will be shown in Section 2.3.

### 2.3. Methods

#### 2.3.1. Holographic Performance Measurement

**Figure 2:**
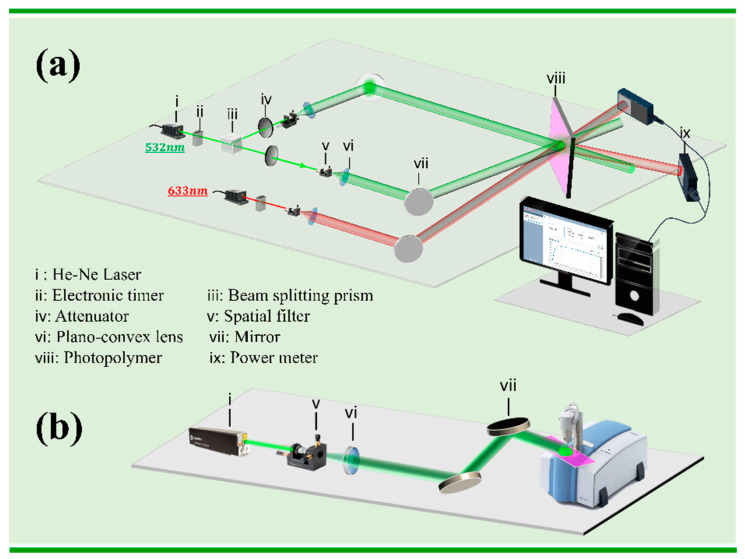
Schematic of experimental test device: (**a**) transmission mode holographic recording setup; (**b**) testing device for real-time infrared spectrum.

In the holographic recording process, the object light and the reference light are coherent and interfere to form a series of interference fringes which, when recorded in the polymer, form gratings. The testing device for photopolymer holographic performance is shown in Figure 2a. The two beams of green light (532 nm) interfere to form interference fringes. Photopolymers convert interference fringes into internal refractive index gratings through polymerization, to achieve information recording. In order to characterize the holographic performance of photopolymers at low spatial frequencies, the angle between the two incident lights is 24°, and the spatial frequency of the grating is about 800 lp/mm. In the experiment, the exposure intensity of green light is 5 mW/cm^2^.

When a beam of red light (633 nm) illuminates the recorded photopolymer at a specific angle (Bragg angle), a beam of diffracted light will be generated. The diffraction efficiency of the material can be obtained by Equation (1):

(1) $$\eta =\frac{I_{d}}{\left(I_{d}+I_{t}\right)}\times 100\%$$

where $I_{d}$ is the intensity of the material diffracted light and $I_{t}$ is the intensity of the transmitted light.

Using the relationship between the Δn and the real-time diffraction efficiency, the real-time refractive index modulation of the material during the exposure process can be obtained. The relationship between refractive index modulation and diffraction efficiency is shown in Equation (2):

(2) $$\Delta n=\frac{\sin^{-1}\left(\eta^{1/2}\right)\cdot \lambda \cdot \cos2\varphi }{\pi \cdot L}$$

where *η* is the diffraction efficiency of the photopolymer; *L* is the thickness of the photopolymer; *λ* is the wavelength of the detection light (633 nm); *φ* is the incident angle of the detection light. In the experiment, *φ* = 14.28°.

Equation (3) is the calculation method of photosensitivity (S):

(3) $$S=\sqrt{\eta_{max}}(V\cdot I_{i}\cdot t)$$

where $\eta_{max}$ and t respectively represent the maximum diffraction efficiency of the photopolymer and the corresponding exposure time; V is the visibility of interference fringes; $I_{i}$ is the intensity of the incident light.

#### 2.3.2. Initial Diffusion Coefficient

The holographic recording of photopolymer involves two important processes, the polymerization of the monomer in the bright area, and the diffusion between the bright and dark areas. In order to study the polymerization and diffusion behavior of monomers in photopolymers, the researchers established a non-local diffusion model (NPDD model) based on the principle of holographic recording. Much work has been carried out in the development of the NPDD model [23,24,25,26]. Monomer diffusion plays an important role in the holographic recording process. It is very important to clarify the value of the diffusion rate of the monomer. Using acrylamide/polyvinyl alcohol (AM/PVA) photopolymers, much research has been conducted on the calculation of diffusion rate values, and researchers have obtained numerous estimation methods [27,28]. The diffusion rate coefficient is the key parameter for characterizing the monomer diffusion rate of the photopolymer.

In this paper, the initial diffusion coefficient ($D_{0}^{*}$) of the monomer in the NPDD model is used to characterize the diffusion rate of NVP. The larger the initial diffusion coefficient, the faster the diffusion rate of the monomer. The calculation method of the initial diffusion coefficient is shown in Equation (4):

(4) $$D_{0}^{*}=\frac{\sin^{-1}\left(\sqrt{\eta_{1}}\right)\cdot \lambda \cdot \cos\theta_{B}\Delta \sqrt{{\bar{\varepsilon }}_{av}}}{\left(n_{m}^{2}-n_{p}^{2}\right)\cdot \pi L\cdot \Delta t_{1}\Delta \varphi_{m0}\cdot V^{2}\cdot K^{2}}\left(1+V\right)$$

where $\eta_{1}$ is the diffraction efficiency at the moment of the initial stage of the reaction; λ is the wavelength of the detection light; ${\bar{\varepsilon }}_{av}$ is the average dielectric constant of the film;$\;n_{m}$ and $n_{p}$ are the refractive index of the monomer and film former, respectively; *L* is the thickness of photopolymer; $\Phi_{m0}$ is the concentration of the monomer before exposure; V is the visibility of the grating; K is the grating vector. Using the diffraction efficiency test device in Figure 2a, the $\eta_{1}$ and $\Delta t_{1}$ at the initial stage of exposure can be obtained. In the experiment, the intensities of the two beams were 5.04 mW/cm^2^ and 5.10 mW/cm^2^.

#### 2.3.3. Real-Time Infrared Spectrum Measurement

According to Lambert Beer’s law, the kinetics of the NVP polymerization of photopolymers during exposure is studied by means of infrared spectroscopy. During the holographic recording process, NVP will polymerize under the initiation of free radicals, which will change the characteristic peak intensity of the C=C bond. The C-H bond ($\delta_{C-H}$) connected to the C=C bond in NVP is selected as the research object. According to the peak intensity of the $\delta_{C-H}$, a curve of the conversion rate of NVP over time can be obtained. The monomer conversion rate is calculated as shown in Equation (5) [16]:

(5) $$\mathrm{CR}=\frac{A_{t}-A_{t=0s}}{A_{t=120s}-A_{t=0s}}$$

where $A_{t}$ represents the intensity of the $\delta_{C-H}$ when the exposure time is *t*.

The polymerization rate of the monomer can be reflected by the slope of the conversion curve. Based on the conversion rate, we can study the difference in the polymerization rate of NVP in the photopolymer.

The infrared spectrum of the photopolymer is measured by the Bruker Alpha II infrared spectrometer (Bruker, Karlsruhe, Germany). The measured wave number is 2000–600 cm^−1^. The test device for real-time infrared spectroscopy is shown in Figure 2b. The exposure intensity is 1 mW/cm^2^ during measurement. The infrared spectrometer performs a spectrum scan every 5 s and the total scan time is 120 s. The ambient temperature is 20 °C, and the relative humidity is 40%.

#### 2.3.4. Simultaneous Thermal Analysis

We dissolved polyvinyl alcohol in deionized water to obtain a PVA solution (5 wt %) and divided it equally into two parts. One of them was named PVA-film, without adding glycerol, and was directly used to prepare the film; the other part was supplemented with 0.02 mol/L glycerol and named Glycerol-PVA. Then, the two abovementioned solutions were used to prepare the film layer separately using the same film-making process. By using the NETZSCH STA 449F5 synchronous thermal analyzer (NETZSCH, Selb, Germany), the physical properties of different polyvinyl alcohol films were studied. The test process is carried out under the protection of N_2_ atmosphere. The heating range is from room temperature to 180 °C, and the heating rate is 10 °C/min.

## 3. Results and Discussion

### 3.1. Effect of Glycerol on Diffusion Rate

**Figure 3:**
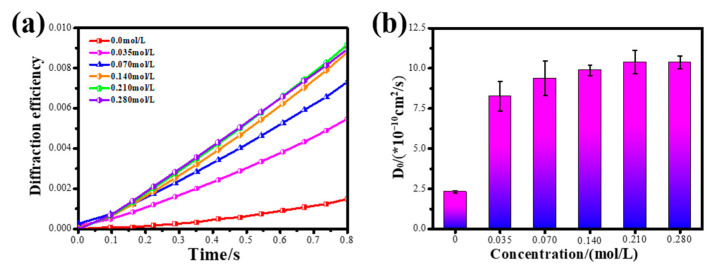
(**a**) The real-time diffraction efficiency of the photopolymer in the initial stage of exposure; (**b**) $D_{0}^{*}$ of NVP in the photopolymer containing different concentrations of glycerol.

Figure 3a shows the real-time diffraction efficiency curve of the photopolymer with or without glycerol at the initial stage of exposure. From Figure 3a, the time difference ($\Delta t_{1}$) of data point collection and the initial diffraction efficiency ($\eta_{1}$) can be obtained. Putting each parameter into Equation (4), we can obtain the initial diffusion coefficient of each photopolymer. The result is shown in Figure 3b.

It can be seen from Figure 3b that the initial diffusion coefficient of NVP in the control group ($D_{0-control\;group}^{*}$) is 2.31 × 10^−10^ cm^2^⁄s; the initial diffusion coefficient of NVP in photopolymers containing glycerol is higher than 8.0 × 10^−10^ cm^2^⁄s. Glycerol successfully improved the diffusion rate of NVP. What is more, as the concentration of glycerol increases from 0.035 mol/L to 0.21 mol/L, the initial diffusion coefficient of NVP in the photopolymer increases from 8.25 × 10^−10^ cm^2^⁄s to 10.38 × 10^−10^ cm^2^⁄s. When the concentration of glycerol continues to increase from 0.21 mol/L to 0.28 mol/L, the initial diffusion coefficient of the monomer is 10.36 × 10^−10^ cm^2^⁄s, which remains basically unchanged.

**Figure 4:**
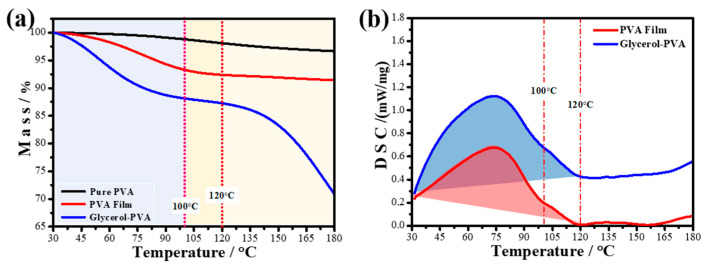
Simultaneous thermal analysis of PVA film with or without glycerol: (**a**) TG; (**b**) DSC.

There may be two reasons why glycerol increases the diffusion rate of NVP in the photopolymer. On the one hand, glycerol acts as a plasticizer, reducing the glass transition temperature of the film-former, which provides more favorable conditions for the diffusion of NVP. On the other hand, the hydroxyl groups of glycerol are easy to combine with water (solvent), which makes the water in the film more difficult to volatilize. More water remains in the film. This is conducive to the extension of the film-forming agent and further reduces the glass transition temperature, which promotes the diffusion of NVP in the film. The thermal analysis experimental results in Figure 4 confirm this conjecture.

From Figure 4a, it can be seen that the quality of pure PVA particles does not change significantly during the process of heating from 30 to 100 °C. However, the polymer film obtained from the PVA solution has a significant mass loss during this heating process. In addition, the DSC curve shows that the photopolymer has a strong and broad endothermic peak in this heating progress. Therefore, it can be inferred that the thermal reaction that occurs during this process is the endothermic volatilization of water in the film. It can be seen from Figure 4a that the mass loss of the PVA film without glycerol is 6.68%, and that of the PVA film with glycerol is 11.87%. Under the same drying conditions, glycerol increases the water content in the photopolymer film, thereby promoting the diffusion of NVP.

As the concentration of glycerol increases, the plasticizing effect increases, making the diffusion of NVP more likely to occur. The monomers in the photopolymers have a higher diffusion rate. However, when the concentration of glycerol is higher than 0.21 mol/L, the plasticizing effect reaches saturation. The diffusion rate of monomers no longer increases with the increase of glycerol concentration.

### 3.2. Effect of Glycerol on Polymerization Rate

**Figure 5:**
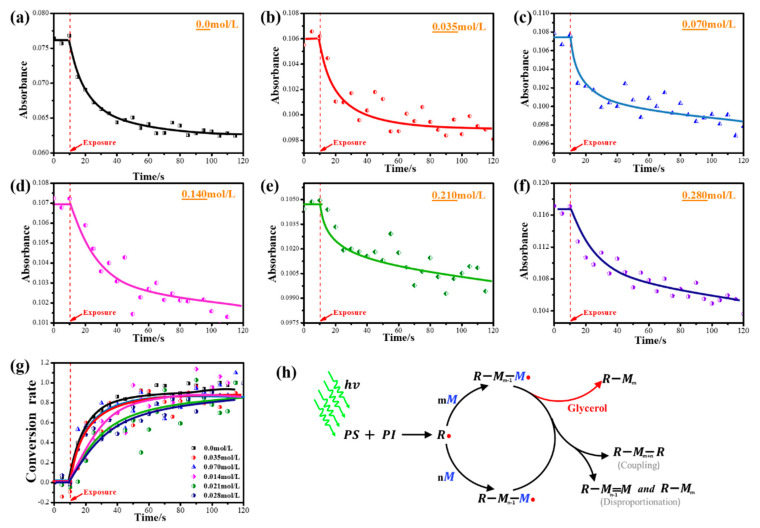
The evolution of the intensity of the $\delta_{C-H}$ (986 cm^−1^) in the photopolymers containing different concentrations of glycerol over time: (**a**) 0.0 mol/L; (**b**) 0.035 mol/L; (**c**) 0.070 mol/L; (**d**) 0.14 mol/L; (**e**) 0.21 mol/L; (**f**) 0.28 mol/L. (**g**) Curve of the conversion rate of NVP in the photopolymers over time. (**h**) Reaction history of the photopolymer. PS and PI are photosensitizer and photoinitiator, respectively; M is monomer; $R^{*}$ is free radical; $R-{\left[M\right]}_{m-1}-M^{*}$ is active polymer chain.

First, the real-time infrared spectroscopy of the photopolymer is measured by the infrared spectrometer. The intensity variation of $\delta_{C-H}$ is obtained from the infrared spectroscopy, as shown in Figure 5a–f. When the exposure starts, the intensity of the $\delta_{C-H}$ will be reduced first, and then stabilize. The relationship between the conversion rate of NVP with exposure time is obtained using Equation (5).

Figure 5g shows the monomer conversion curve of each photopolymer. The polymerization rate of the monomer can be reflected by the slope of the conversion curve. A larger slope indicates that the photopolymer has a larger polymerization rate. It is obvious from Figure 5g that when the concentration of glycerol is relatively low, the polymerization rate of NVP does not decrease to a large extent; but when the concentration of glycerol is higher than 0.14 mol/L, the polymerization rate of NVP is significantly decreased. The glycerol changed the polymerization rate of NVP in the photopolymer, possibly because it acts as a free radical scavenger during the reaction [13].

Figure 5h shows the reaction history of the photopolymer under exposure. The photosensitizer and the photoinitiator react to generate a primary radical ($R^{*}$) under the excitation of a laser of a specific wavelength. The reactive primary radical will initiate the polymerization of NVP to form an active polymer chain ($R-{\left[M\right]}_{m-1}-M^{*}$) with a certain chain length. The active polymer chain can continue to initiate the polymerization of NVP until the two active chains collide and undergo a coupling or disproportionation reaction. Eventually, both active chains lose polymerization activity and produce polyvinylpyrrolidone (PVP) with a certain chain length.

When glycerol was added to the photopolymer, the active polymer chain directly generates PVP because of the reaction with glycerol, as shown in Figure 5h. This reaction reduces the life of the active polymer chain and decreases the concentration of free radicals, inhibiting the polymerization of NVP initiated by the active polymer chain. The polymerization rate of the monomer is directly proportional to the concentration of the monomer and free radicals ($R_{P}\propto \left[M\right]\left[R\cdot \right]$). Therefore, the polymerization rate of NVP decreased after glycerol was added. As the concentration of glycerol increases, more active polymer chains may be terminated and lose polymerization activity, so that the concentration of free radicals in the material is further reduced. Therefore, the polymerization rate of NVP gradually decreased. However, the free radical scavenging effect is limited by the concentration of glycerol. At low concentrations ($c_{glycerol}\;$< 0.14 mol/L), the scavenging effect is not obvious.

The faster polymerization rate of the monomer means that the monomer can form a concentration gradient faster between the light and dark areas. In turn, the diffusion process of the monomer is driven. Moreover, the faster the polymerization rate, the greater the concentration gradient between light and dark areas. This is more conducive to the enrichment of the monomer in the bright region and the formation of a refractive index grating. Therefore, in terms of polymerization rate, glycerol reduces the polymerization rate of NVP, which is not conducive to improving the holographic performance of the photopolymer.

### 3.3. Effect of Glycerol on Holographic Performance

**Figure 6:**
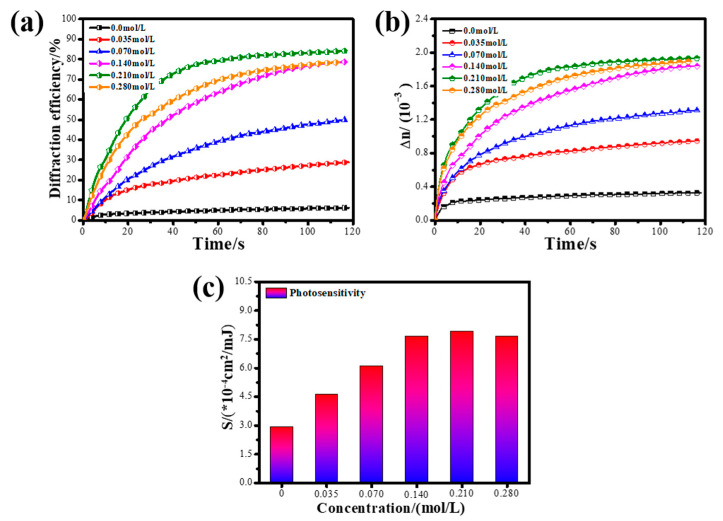
Effect of glycerol on the holographic performance of photopolymers: (**a**) curve of real-time diffraction efficiency; (**b**) curve of refractive index modulation; (**c**) photosensitivity.

Figure 6 shows the holographic properties of photopolymers containing different concentrations of glycerol. The concentrations of glycerol are 0 mol/L, 0.035 mol/L, 0.07 mol/L, 0.14 mol/L, 0.21 mol/L and 0.28 mol/L, respectively. According to Figure 6, it can be seen that the maximum diffraction efficiency of the control group without glycerol is only 6.9%, the maximum refractive index modulation (Δn) is 3.43 × 10^−4^ and the photosensitivity is 2.92 × 10^−4^ cm^2^/mJ. When the glycerol was added to the photopolymer, the maximum diffraction efficiency, maximum refractive index modulation and photosensitivity of the photopolymer all increase. The reason why glycerol improves the holographic performance of photopolymers is that glycerol changes the formation process of the internal refractive index grating.

**Figure 7:**
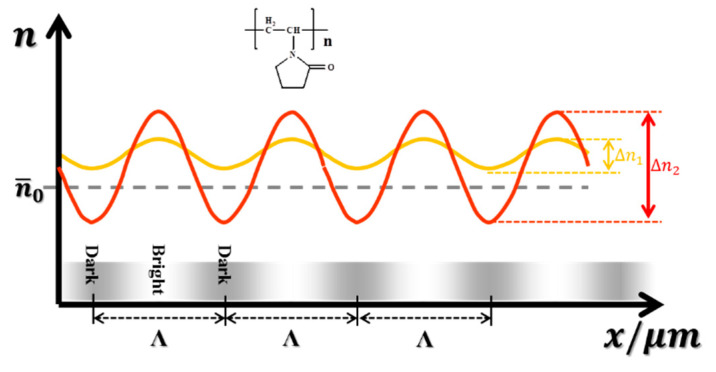
Schematic diagram of the internal refractive index distribution of photopolymer. ${\bar{n}}_{0}$ is the average refractive index of the unexposed photopolymer material; Λ is the grating period; the yellow line represents the photopolymer without glycerol; the red line represents the photopolymer with glycerol.

Figure 7 shows the schematic diagram of the internal refractive index distribution of photopolymer. We assume that the components in the photopolymer are uniformly distributed before exposure, and the internal refractive index is equal everywhere. Its average refractive index is ${\bar{n}}_{0}$, as shown by the dashed line in Figure 7.

The results of Section 3.2 indicate that monomers in the photopolymer without glycerol have a high polymerization rate, so a great deal of polyvinylpyrrolidone (PVP) can be quickly enriched in the bright area, and the refractive index of the bright area ($n_{\mathrm{bright}}$) increases at the initial stage of exposure. At this time, $n_{\mathrm{bright}}$> ${\bar{n}}_{0}$. The active polymer chains will undergo reverse diffusion driven by the concentration gradient. However, Section 3.1 shows that the diffusion rate of NVP in the photopolymer without glycerol is low. A large number of monomers that cannot diffuse to the bright regions will directly polymerize in the dark regions under the initiation of the active polymer chains. The PVP is generated in the dark regions, which increases the refractive index of the dark regions ($n_{\mathrm{dark}}$). As a result, the difference in refractive index between the bright and dark regions of the photopolymer is reduced. Finally, a refractive index grating with low refractive index modulation ($\Delta n_{1\;}$=$\;n_{\mathrm{bright}}-n_{\mathrm{dark}}$) is formed, as shown by the yellow line in Figure 7. From the aforementioned experimental results, it is known that glycerin will react with the active polymer chains, reducing the concentration of active polymer chains in the material. As a result, the concentration gradient of the active polymer chains between the bright regions and the dark regions is reduced, which weakens the reverse diffusion of the active polymer chains. The glycerol, added to the photopolymer, reduces the reverse diffusion of the active polymer chains and increases the diffusion rate of NVP, which allows more monomers to diffuse to the bright regions to polymerize. In this process, the $n_{\mathrm{dark}}$ will decrease due to the diffusion of the monomer to the bright regions ($n_{\mathrm{dark}}<{\bar{n}}_{0}$). Compared with the photopolymer without glycerol, the composition difference between the bright and dark regions inside the material increases. A refractive index grating with greater refractive index modulation ($\Delta n_{2}$) is formed, as shown by the red line in Figure 7. Therefore, compared with the control group, the photopolymer containing glycerol showed higher photosensitivity, maximum diffraction efficiency and refractive index modulation.

In addition, the rapid diffusion of the monomer during the holographic recording process will increase the concentration of the monomer in the bright regions, thereby increasing the polymerization rate. Therefore, after glycerol is added, the improvement of the diffusion rate weakens the influence of the polymerization rate on the formation of the refractive index grating. The diffusion rate of the monomer becomes the key factor affecting its modification effect.

Therefore, the holographic performance of the photopolymer increases with the concentration of glycerol from 0.035 mol/L to 0.21 mol/L due to the increase in the diffusion rate of NVP, as shown in Figure 6. The $\eta_{max}$ gradually increased from 28.86% (0.035 mol/L) to 84.1% (0.21 mol/L); the Δn increased from 0.95 × 10^−3^ to 1.95 × 10^−3^; and the photosensitivity increased from 4.62 × 10^−4^ cm^2^/mJ to 7.91 × 10^−4^ cm^2^/mJ. When the concentration continued to increase from 0.21 mol/L to 0.28 mol/L, the holographic performance of the photopolymer did not improve, but basically remained stable. The reason for this result is that the polymerization rate and diffusion rate remain basically stable in this concentration range. In the experiment, we found that the photopolymer film will shrink seriously during the drying process when the concentration of glycerol is higher than 0.21 mol/L. Such photopolymers cannot be used for performance testing and holography.

In summary, when the concentration of glycerol is 0.21 mol/L, the photopolymer has an excellent holographic performance. The $\eta_{max}$, Δn and photosensitivity of the photopolymer is 84.1%, 1.95 × 10^−3^ and 7.91 × 10^−4^ cm^2^/mJ, respectively. Compared with the control group, the maximum diffraction efficiency, maximum refractive index modulation and photosensitivity at low spatial frequencies (800 lp/mm) increased by 11.19 times, 4.69 times and 1.71 times, respectively.

### 3.4. Transmission Holographic Recording and Reproduction

**Figure 8:**
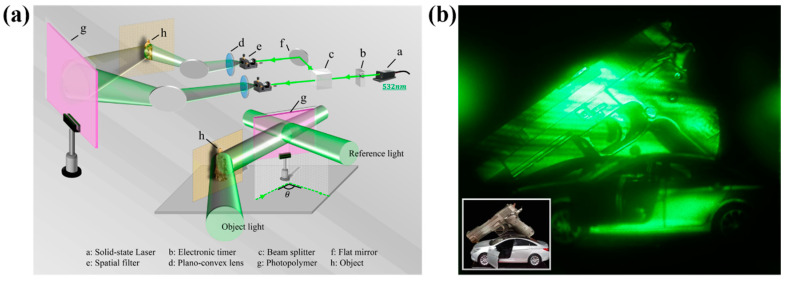
(**a**) Schematic diagram of transmission holographic device; (**b**) reproduction image of transmission hologram using green light.

The photopolymer modified by glycerol ($c_{glycerol}\;$= 0.21 mol/L) was selected for transmission holographic recording and reproduction. The schematic diagram of the transmissive holographic printing device is shown in Figure 8a. The ratio of object light to reference light is 1:1. The angle between the two beams is 24.2°. Figure 8b shows the transmission hologram reproduced by green light. The reproduced image has high image brightness. Moreover, the details of the object are very clear. It can be seen that the modified photopolymer can be successfully applied to transmission holographic recording at low spatial frequencies.

## 4. Conclusions

The addition of glycerol effectively solves the problem that NVP is difficult to diffuse at low spatial frequencies. At a concentration of 0.21 mol/L, the initial diffusion coefficient increased to 10.38 × 10^−10^ cm^2^⁄s, which is 4.5 times that of the unmodified photopolymer. The experimental results show that the increase in the diffusion rate of NVP is the key reason for the modified photopolymer. When the NVP-based photopolymer is modified with glycerol, its diffraction efficiency, refractive index modulation and photosensitivity at a low spatial frequency (800 lp/mm) have been improved. As the concentration of glycerol increases, the holographic performance first increases and then remains stable. The optimal concentration of glycerol is 0.21 mol/L. At this concentration, the maximum diffraction efficiency of the photopolymer is 84.1%, the refractive index modulation is 1.95 × 10^−3^, and the photosensitive sensitivity is 7.91 × 10^−4^ cm^2^/mJ. Compared with the control group, the maximum diffraction efficiency, maximum refractive index modulation and photosensitivity at low spatial frequencies (800 lp/mm) have increased by 11.19 times, 4.69 times and 1.71 times, respectively. Using the optimized photopolymer for transmission holographic printing and reproduction, we have obtained a clear and bright transmission hologram. The photopolymer modified with glycerol is expected to find applications in fields such as holography and diffractive optics.
